# Supplementary material for: Neuroprosthetic Decoder Training as Imitation Learning
Source: PLoS Comput Biol. 2016 May 18;12(5):e1004948. doi: 10.1371/journal.pcbi.1004948 (PMC4871564; doi:10.1371/journal.pcbi.1004948)
Supplement: S1 Text — We restate and interpret the theoretical results for DAgger. We also describe specific bounds on regret for selected BCI update rules under a quadratic loss and linear decoder. (PDF) [file pcbi.1004948.s001.pdf]

Supporting Text

DAgger guarantees

A major contribution of the DAGGER meta-algorithm for the imitation learning setting is that the loss can be bounded in relation to online learning regret bounds [1]. This allows the analysis of DAGGER through established results in the literature. Note that d_π is the distribution over states that arise from policy/decoder π.

Theorem 1 (modified statement of Theorem 4.1 from [1])**.** *For* DAGGER*, there exists a policy* $\hat{\pi} \in \pi^{(1)}, \dots \pi^{(K)}$ *s.t.*
$\mathbb{E}_{\mathbf{x},\mathbf{n},\mathbf{o}\sim d_{\hat{\pi}}}[\ell(\hat{\pi}(\mathbf{x},\mathbf{n}),\mathbf{o})] \leq \epsilon_K + \frac{1}{K}\cdot(2\ell_{\max})\left[T\sum_{k=1}^{K}\beta_k\right] + \gamma_K$*, where* γ_K *is the average regret of* $\pi^{(1)}, \dots \pi^{(K)}$*.*

This theorem states that the expected loss of the policy (i.e. decoder) resulting from DAGGER is upper bounded by three terms. The first and second terms depend on the model and meta-algorithm settings, whereas γ_K will depend on the specific update method chosen. We note as well that this assumes that T is an upper bound on $T_1, \dots, T_K$, so that the length of any trajectory is less than the maximum duration T.

We first discuss the two terms shared across update rules. The first term:

$$\epsilon_K = \min_{\pi\in\Pi}\sum_{k=1}^{K}\mathbb{E}_{\mathbf{x},\mathbf{n},\mathbf{o}\sim d_{\pi^{(k)}}}[\ell(\pi(\mathbf{x},\mathbf{n}),\mathbf{o})], \tag{1}$$

is the loss that would have been incurred if the best decoder had been used the whole time. In some settings ϵ_K may approach 0, but typically there is observation noise (i.e. neural noise in this case), and additional variability could arise from model mismatch. For BCI, we consider ϵ_K to be the error due to neural noise of the best decoder. The second term is dependent on β_i, which controls how the algorithm blends the oracle policy and the learned decoder during training. β_i can be chosen to be 0 for all i, which would eliminate this term. However, this term decays quickly, $\mathcal{O}(\frac{1}{K})$, so assisted training may help performance in practice. The constant $\ell_{\max}$ is the maximum value of $\ell(\pi(\mathbf{x},\mathbf{n}),\mathbf{o})$ for $\pi \in \pi^{(1)}, \dots, \pi^{(K)}$ and $k \in 1, \dots, K$ over effector states and neural observations. In the BCI setting, degrees of freedom of the effector have a bounded range and neural activity is physiologically bounded. So we expect $\ell_{\max}$ will scale with the variance of the decoded variable.

The γ_K term, which is the average regret of the $\pi^{(1)}, \dots \pi^{(K)}$, is given by

$$\gamma_K = \mathrm{Regret}_K(\Pi)/K. \tag{2}$$

We note that Theorem 1 covers the asymptotic case. Theorem 4.2 of [1] addresses the finite sample case, where the bound will hold with probability at least δ when the term $\ell_{\max}\sqrt{\frac{2\log(1/\delta)}{K}}$ is added to the right hand side. The effect of this is that it adds a small amount of slackness to Theorem 1 that increases if we want the theorem to hold with higher probability. This term decays $\mathcal{O}\left(\frac{1}{\sqrt{K}}\right)$. While this term may have a slower rate than the other terms, we note both that this will still maintain a no-regret algorithm and that for practical values this term will be comparatively small.

As written in [1], Theorem 1 requires the use of probabilistic mixing of the oracle policy and the learned policy. Probabilistic mixing refers to using the oracle policy exactly with probability β_k and the decoded policy exactly with probability $1-\beta_k$. However, we have advocated using a linear mixture of the decoding policy rather than probabilistic mixing to match the existing BCI literature. We note that this will only superficially alter the form of Theorem 1. The term $\frac{1}{K}\cdot(2\ell_{\max})\left[T\sum_{k=1}^{K}\beta_k\right]$ is

dependent on Lemma 4.1 of [1], but a slightly different bound can be derived using triangle inequality (the naive bound given in Lemma 4.1 of [1]). When using this linear mixing, the bound in Theorem 1 would instead have a term $\frac{1}{K} \cdot (2\ell_{\max}) \left[\sum_{k=1}^{K} 1_{\{\beta_k \neq 0\}}\right]$, where $1_{\{\cdot\}}$ denotes an indicator function (1 if the condition is true, 0 otherwise). This term is the same for any non-zero β_k chosen. This is a very loose bound, but the original bound is also very loose on this term and for the sequences of β_k used in the experiments there will be minimal differences. For example, the sequence of $\beta_1 = 1$ and $\beta_k = 0,\ k > 1$ will yield the same result on this term for linear mixing and probabilistic mixing.

Regret in linear-quadratic setting

For a given objective function, we can use established results to analyze the decoder update options. We specialize our statements for the case of a quadratic loss. The SSKF takes a linear autoregressive form, so concrete statements about the quadratic loss will be applicable for the SSKF decoder. We consider a linear decoder which attempts to estimate intention from covariates – we let $\mathbf{W}$ be the parameters of the linear decoder π and $\mathbf{z}_{kt}$ be the covariates. With a slight abuse of notation, we define:

$$\ell(\mathbf{W}, \mathbf{z}_{kt}, \mathbf{o}_{kt}) = ||\mathbf{W}\mathbf{z}_{kt} - \mathbf{o}_{kt}||^2. \tag{3}$$

This generic linear decoder may be explicitly specialized to the SSVKF in Eqn. 8 by setting $\mathbf{W} = [\mathbf{F}_v\ \mathbf{b}_v\ \mathbf{G}_v]$ and $\mathbf{z}_{kt} = [\mathbf{n}_{kt}\ \mathbf{1}\ \mathbf{x}_{kt}]^\top$.

The linear-quadratic setting is widely seen in applications and mean square error convergence properties of linear models have been analyzed specifically for the least mean square (LMS) algorithm [2]. Perhaps surprisingly, the quadratic loss does not satisfy the assumptions required by the simplest online optimization frameworks for regret analysis, because the total loss is not a Lipschitz function [3]. An L-Lipschitz function is defined as $|f(\mathbf{x} + \delta) - f(\mathbf{x})| \leq L||\delta||$ with respect to a given norm, typically the ℓ_2 norm (i.e. $||\mathbf{x}||_2 = \left(\sum_i x_i^2\right)^{1/2}$). For a squared loss, L would go to infinity at the tails. However, the squared loss is a Lipschitz function over a bounded region, so in practical settings this is sufficient.

We consider the three updates in Table 1 (OGD, FTL, and MA), specifically for the linear-quadratic case. The OGD update takes the form:

$$\mathbf{W}_{k+1} = \mathbf{W}_k - \frac{1}{\eta_k} \nabla_{\mathbf{W}} \sum_{t=1}^{T_k} \ell(\mathbf{W}_k, \mathbf{z}_{kt}, \mathbf{o}_{kt}). \tag{4}$$

The regret scales as $\mathcal{O}(\sqrt{K})$, so γ_K is $\mathcal{O}(\frac{1}{\sqrt{K}})$ [4].

Strong convexity on the loss $\sum_{t=1}^{T_k} \ell(\mathbf{W}_k, \mathbf{z}_{kt}, \mathbf{o}_{kt})$ for all k will give a regret rate of $\mathcal{O}(\log K)$ [5]. This can be achieved by adding regularization on $\mathbf{W}$, which is typically done in practice. Alternatively, this condition will usually be satisfied when T_k is greater than the number of parameters in $\mathbf{W}_k$, although this also depends on the data $\mathbf{z}_{kt}$ and $\mathbf{o}_{kt}$.

The MA update is given by:

$$\mathbf{W}_{k+1} = \lambda \mathbf{W}_{\mathbf{k}} + (1 - \lambda)\mathbf{W}_k^* \tag{5}$$

$$\mathbf{W}_k^* = \arg\min \sum_{t=1}^{T_k} \ell(\mathbf{W}_k, \mathbf{z}_{kt}, \mathbf{o}_{kt})) \tag{6}$$

for $\lambda \in [0, 1]$. As discussed in the main text, this algorithm suffers from regret that is at least $O(K)$, so it is not a no-regret algorithm.

The FTL update is given by:

$$\pi^{(k)} = \arg\min_{\pi \in \Pi} \sum_{k'=1}^{k-1} \sum_{t=1}^{T_k} \ell(\pi, \mathbf{z}_{k't}, \mathbf{o}_{k't}), \quad (7)$$

$$\Leftrightarrow \quad \mathbf{W} = \arg\min_{\mathbf{W}} \sum_{k'=1}^{k-1} \sum_{t=1}^{T_{k'}} ||\mathbf{W}\mathbf{z}_{k't} - \mathbf{o}_{k't}||^2 + g(\mathbf{W}). \quad (8)$$

$g(\mathbf{W})$ is a optional regularization penalty. Typical regret analysis for FTL depends on the Lipschitz properties of the loss function (for a smooth function, this implies that the gradient is bounded). We emphasize that any standard optimization technique can be used here, and the FTL strategy is not sensitive to step-sizes. Restricting the parameter set to a ball such that $||\mathbf{W}||_2^2 \leq B$ and assuming $||\mathbf{z}_{kt}||_2 \leq 1 \; \forall k, t$ and $||\mathbf{o}_{kt}||_2 \leq 1 \; \forall k, t$ yields $\mathcal{L}(W, \mathcal{D}^{(1:k)}) = \sum_{k'=1}^{k-1} \sum_{t=1}^{T_{k'}} \ell(\mathbf{W}, \mathbf{z}_{k't}, \mathbf{o}_{k't})$ as B^2-Lipschitz, which can be used to analyze a bounded least-squared problem [5]. These conditions will be satisfied when using feasible data generated in the system with a regularized $g(\mathbf{W})$.

To get a better regret bound for FTL, we must analyze it through the perspective of another approach, called *Exponentially-weighted online optimization* (EWOO) [5]. We will not talk about this method in general; however, for the specific case of the least squares loss function and ℓ_2 regularization, the updates for EWOO are identical to the updates for FTRL (or FTL if the regularization is omitted). We emphasize that these updates are not equivalent in general. This is beneficial in our case because it derives a logarithmic regret bound $\mathcal{O}(\frac{\ell_{\max}}{\alpha} \log K)$, and α will be described below. In this case, γ_K will scale as $\mathcal{O}(\log(K)/K)$, which is an improvement over the $\mathcal{O}(1/\sqrt{K})$ rate. Instead of being dependent on Lipschitz smoothness of the loss function, the constants are dependent on an alternative property called *α-exp-concavity*, which is defined:

$$\forall \mathbf{W} \in \mathcal{P}, \forall k \in 1, \ldots, K : \nabla^2[\exp(\alpha \mathcal{L}(W, \mathcal{D}^{(1:k)}))] \preceq 0. \quad (9)$$

This property depends on a non-empty convex set, $\mathcal{P} \subseteq \mathbb{R}^P$, which corresponds to the *feasible* parameters of the decoder/policy. In general, this is unconstrained; however, given certain properties of the dataset, the set $\mathcal{P}$ can be quite constrained. We note that any strongly convex function has α-exp-concavity, but that this is a *weaker* property than strong convexity. For the least squares problems, without this assumption the α constant can be arbitrarily bad. We will discuss reasonable assumptions below, and mention how they restrict $\mathcal{P}$ and therefore the constant α as well.

Since α affects the regret of the algorithm, we need to get a sense of the value of α in practice in order to truly assess the performance of this algorithm. For our case, α can be simplified to $\frac{1}{\alpha} = \max_{\mathbf{W} \in \mathcal{P}, k, t} ||\mathbf{W}\mathbf{z}_{kt} - \mathbf{o}_{kt}||_2^2$. Next, we will utilize a standard trick, where $\mathcal{P}$ is set and analyzed over realizable values that $\mathbf{W}$ can take [3]. To get a simpler form of this analysis, we will make the significant, but reasonable, assumption that the worst parameter settings will be our initialization, which we set to $\mathbf{0}$ here for simplicity. This assumption makes $\frac{1}{\alpha}$ scale as $\mathcal{O}(||\mathbf{o}||_2^2)$. The same assumption will set $\ell_{\max}$ to $\mathcal{O}(||\mathbf{o}||_2^2)$. Hence, we expect the total regret to scale as $\mathcal{O}(||\mathbf{o}||_2^4 \log K)$. This gives a strong, but reasonable, dependency on the magnitude of the oracle movements.

References

1. Ross S, Gordon GJ, Bagnell JA. A Reduction of Imitation Learning and Structured Prediction to No-Regret Online Learning. Artificial Intelligence and Statistics (AISTATS). 2011;15.

2. Widrow B, Stearns SD. Adaptive signal processing. Englewood Cliffs. 1985;.

3. Shalev-Shwartz S. Online Learning and Online Convex Optimization. Foundations and Trends in Machine Learning. 2011;4(2):107–194.

4. Kivinen J, Warmuth MK. Additive Versus Exponentiated Gradient Updates for Linear Prediction. In: Proceedings of the Twenty-seventh Annual ACM Symposium on Theory of Computing. STOC '95. New York, NY, USA: ACM; 1995. p. 209–218.

5. Hazan E, Agarwal A, Kale S. Logarithmic regret algorithms for online convex optimization. Machine Learning. 2007;69(2-3):169–192.
