# Supplementary figures and images for: Neuroprosthetic Decoder Training as Imitation Learning
Source: PLoS Comput Biol. 2016 May 18;12(5):e1004948. doi: 10.1371/journal.pcbi.1004948 (PMC4871564; doi:10.1371/journal.pcbi.1004948)

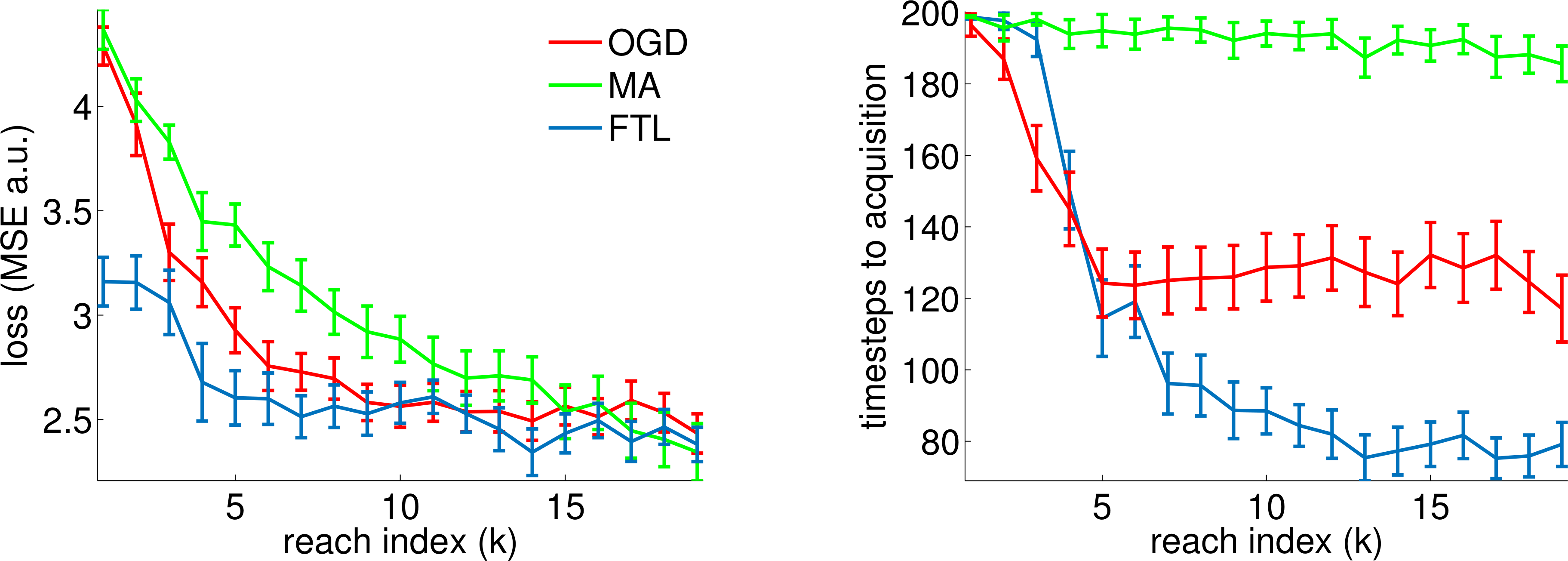

Supplement: S1 Fig — This figure compares MSE and time to acquisition for the cursor task, and motivates the use of SSE in the figures in the main text. Left panel depicts MSE for cursor task (for same trials as SSE curves in Fig 2). Right panel depicts time to acquisition for the same set of trials. While we might hope that MSE would give a complete indication of performance, this is not the case. This is because the quality of the different algorithms are differentially reflected when considering trial duration. Low MSE can be achieved multiple different ways—essentially mapping to the bias-variance tradeoff. In the trials considered here, the slow acquisition for the MA decoder arises from bias towards decoder outputs with smaller magnitude. (TIFF) [file pcbi.1004948.s002.tiff]
